# Exploring Perspectives and Barriers in Implementing Home Monitoring for Pediatric Pulmonary Diseases: Mixed Methods Study

**DOI:** 10.2196/101452

**Published:** 2026-10-20

**Authors:** Mees HP Stoop, Romy Krul, Badies HAN Manai, Iris Groothuis, Arwen J Sprij, Marianne Nuijsink, Hettie M Janssens, Matthijs D Kruizinga

**Affiliations:** 1 Department of Pediatrics Haga Teaching Hospital Juliana Children's Hospital The Hague, South Holland The Netherlands; 2 Department of Pediatrics MosaKids Children’s Hospital Maastricht University Medical Centre Maastricht, Limburg The Netherlands; 3 Department of Pediatrics Sophia Children’s Hospital Erasmus MC Rotterdam, South Holland The Netherlands; 4 Cystic Fibrosis Core Network Group ERN LUNG Frankfurt, Hesse Germany; 5 Department of Pediatrics Leiden University Medical Center Leiden, South Holland The Netherlands

**Keywords:** telemedicine, child, asthma, cystic fibrosis, patient perspectives, patient acceptance of health care, caregiver perspectives, digital health

## Abstract

**Background:**

Home monitoring has the potential to enable early detection of disease progression in pediatric lung diseases such as asthma and cystic fibrosis (CF). There is minimal evidence regarding the opinions of caregivers and children on home monitoring.

**Objective:**

This study aimed to explore children’s and caregivers’ perspectives on home monitoring.

**Methods:**

This multicenter, mixed methods study included children aged 6 to 18 years with asthma or CF and their caregivers. Participants were recruited from Juliana Children’s Hospital and Erasmus MC Sophia Children’s Hospital between September 2023 and June 2025. Participants completed a structured questionnaire assessing experiences, attitudes, and potential barriers and facilitators to home monitoring. Fourteen children and their caregivers took part in in-depth interviews. Conventional content analysis was applied to both open-text and interview data.

**Results:**

A total of 113 caregiver-child dyads (62 [54.9%] asthma and 51 [45.1%] CF) participated in this study. Willingness to use home monitoring was high among both children (asthma: n=47, 83.9%; CF: n=30, 62.5%) and caregivers (asthma: n=54, 87.1%; CF: n=44, 86.3%). Children with asthma and their caregivers showed significantly greater willingness to use the proposed types of home monitoring than children with CF and their caregivers. Caregivers who expressed difficulty with symptom interpretation were significantly more motivated to use home monitoring. Key barriers included time demands, usability, device size, and concerns about maintaining personal contact with health care providers. Facilitators included compact, user-friendly devices, clear instructions, and improved disease understanding.

**Conclusions:**

High willingness to adopt home monitoring was observed among children with asthma and children with CF, but preferences and perceived barriers differ. Tailoring device choice and implementation strategies to patient needs, minimizing burden, and integrating medical oversight are important for successful uptake.

## Introduction

Asthma and cystic fibrosis (CF) are chronic diseases in the pediatric population, characterized by recurrent pulmonary exacerbations that may lead to hospital visits and admissions. These exacerbations are associated with a higher perceived disease burden, a decline in pulmonary function, and higher health care costs [1]. Effective control of asthma and CF is crucial for reducing the clinical burden and adverse outcomes associated with exacerbations. Ideally, it would be possible to predict pulmonary exacerbations to start treatment in a timely manner before symptoms have progressed. Remote health care, digital biomarkers, and home monitoring offer the potential to recognize exacerbations early, minimizing both acute complications and long-term damage while also reducing health care costs [2].

In the pediatric population, assessing disease activity and predicting exacerbations are especially challenging. Young children find it difficult to perceive and express their symptoms, causing difficulty in parental recognition of symptoms [3], which home monitoring may help alleviate [4-9]. Real-time data may be necessary to effectively monitor disease activity using objective measurements. Devices such as smartwatches, spirometers, and other home monitoring devices have been used previously. However, the compliance of patients significantly decreases when the duration and frequency of home monitoring assessments increase [10]. In contrast, evidence suggests that patient empowerment and patients’ capacity to understand and interpret their health-related data facilitate sustained engagement with digital health tools [11]. Despite these findings, most studies on home monitoring primarily focus on clinically relevant outcomes, while the patient perspective, particularly the pediatric perspective, is underrepresented [12]. It is essential to understand children’s and caregivers’ views and motivations regarding home monitoring tools to successfully implement them. Therefore, this exploratory study aims to assess the perspectives of children and caregivers regarding home monitoring and to identify barriers and facilitators to its use and implementation. In addition, preferences for different home monitoring methods and devices are investigated, as well as the circumstances under which home monitoring would be considered acceptable.

## Methods

### Study Design

This study was conducted at Juliana Children’s Hospital (Haga Teaching Hospital, The Hague, the Netherlands) and Erasmus MC Sophia Children’s Hospital (Rotterdam, the Netherlands). Data were collected between September 2023 and June 2025.

### Ethical Considerations

This study was submitted to the Medical Ethics Committee Leiden Delft Den Haag (Zuid-Holland, the Netherlands), which concluded that the study did not fall under the Dutch Medical Research Involving Human Subjects Act (Wet Medisch-Wetenschappelijk Onderzoek Met Mensen [WMO]). The study protocol was reviewed and approved by the institutional review board of the Haga Teaching Hospital (T23-050). Written informed consent was obtained from caregivers and children aged ≥12 years, while children aged <12 years provided verbal assent.

### Recruitment

Participants were recruited from the outpatient clinics and inpatient wards to include children with different disease severities. Participants were informed about the study by their treating pediatrician. If they were interested in participating, the researcher subsequently provided further information about the study. Eligible participants were aged 6 to 18 years with a confirmed diagnosis of asthma (per Global Initiative for Asthma [GINA] guidelines) or CF (confirmed by genetic testing). The only exclusion criterion was insufficient proficiency in Dutch, based on the caregiver’s indication that the child was able to understand the questionnaire independently. A formal power calculation was not performed due to the exploratory nature of the study. The target sample of 120 participants (60 with asthma and 60 with CF) was considered sufficient to allow meaningful subgroup comparisons and thematic saturation.

### Study Procedures

This study followed a convergent mixed methods design, combining quantitative survey data and qualitative interviews to explore and contextualize participants’ perspectives. The study was reported in accordance with the Good Reporting of A Mixed Methods Study (GRAMMS) recommendations (Multimedia Appendix 1) [13]. The mixed methods approach was used to quantify preferences regarding home monitoring while also exploring the underlying reasons, barriers, and facilitators that could not be fully captured through predefined questionnaire responses alone. Participants were asked to complete a structured questionnaire, developed for this study, assessing experiences and attitudes toward home monitoring. At the start of the study, participants were also invited to participate in a semistructured interview to explore these themes in greater depth after completing the questionnaire. Completing the questionnaire took approximately 30 minutes and consisted of 2 sections. One section was to be completed by the caregiver first, and subsequently, a shorter section was completed by the child. Questionnaires were administered using multiple methods to accommodate participant preferences: electronically or on paper. The digital questionnaire could also be completed at home; the paper questionnaire was completed in the hospital. Research staff were available for assistance.

Incomplete caregiver questionnaires were excluded from analysis at the case level. The first item of the pediatric questionnaire asked caregivers whether their child was able to understand and answer the questions independently. This was included to account for differences in age and level of comprehension among the participating children. Participants were not excluded based on this item because caregiver-reported data were available regardless of whether the child was able to complete the questionnaire.

### Instruments

The questionnaire (Multimedia Appendix 2) was specifically developed for the purpose of this study and was not based on a previously validated instrument. The questionnaire was reviewed and informally pretested by members of the research team, laypersons, children, parents, and medical specialists to assess the quality of the questions, ensuring they did not imply or use unclear terminology that could potentially influence the participants. On the basis of several rounds of feedback, the wording and structure of the questions were refined before study initiation.

Most questions had a 5-point Likert scale outcome or a yes or no outcome. All questions had a text box option to elaborate on the answers provided. The questionnaire consisted of 5 categories. The first category included demographic and clinical characteristics of the participants. The second category assessed whether caregivers had prior exposure to digital monitoring tools. The third category explored caregivers’ attitudes toward various monitoring devices, including smartwatches, lung function meters, cough detectors, smart inhalers, air quality monitors, medication reminders, and symptom questionnaires. A smart inhaler is a small electronic device attached to an inhaler that automatically detects and time stamps inhaler use. These monitoring devices were selected based on previous literature [14-18]. The fourth category focused on the conditions under which caregivers would consider using such devices. The final category included open-ended questions about potential barriers and facilitators to the implementation of home monitoring systems.

The semistructured interviews addressed the themes mentioned in the questionnaire and were conducted by 1 researcher. The interviews were audio-recorded and transcribed verbatim by 2 researchers without the use of transcription software. Member checking was not performed. Data saturation was reached after 14 interviews.

### Data Analysis

All analyses were performed using RStudio (version 4.4.2; R Foundation for Statistical Computing). Participant characteristics were analyzed descriptively. To summarize participants' overall attitude toward home monitoring, a cumulative device score was calculated. For each proposed monitoring modality, willingness to use the device was scored as +1, unwillingness as –1, and a neutral or “no opinion” response as 0. Neutral and “no opinion” responses were both assigned a value of 0 because neither reflects a definitive driver of, nor a barrier to, device adoption. All modalities were weighted equally to capture broad technological acceptance, rather than being weighted by patient burden or clinical utility, which was beyond the scope of this exploratory measure. A score of –7 means that a participant had a negative attitude toward every type of device, and a score of +7 means that a participant had a positive attitude toward all devices. The children with CF and their caregivers could reach a maximum of 6 points, as most children with CF did not use inhalers. No normalization of the cumulative device score was performed.

Normally distributed variables are reported as mean (SD), and nonnormally distributed variables are reported as median (IQR). Categorical variables were summarized as absolute numbers and percentages. Comparative analyses were performed to assess differences between the asthma and CF groups. Likert scale questions were analyzed using the Mann-Whitney *U* test. Categorical variables were compared between the asthma and CF groups using the chi-square test. Associations between ranked variables were evaluated with the Spearman rank correlation coefficient. A 2-sided *P*<.05 was considered statistically significant. As this was an exploratory study, no correction for multiple testing was applied.

Qualitative open-text responses and interview transcripts were analyzed using conventional content analysis to identify key barriers and facilitators to home monitoring adoption [19]. The data were coded inductively without the use of a predefined coding framework. As new concepts emerged from the data, new descriptive codes were created, and similar codes were grouped into broader categories representing the main barriers and facilitators. For both data sources, 1 researcher performed the initial analysis, and a second researcher subsequently reviewed the data, coding, and resulting interpretation. The final coding framework and categories were then discussed within the research team and refined where appropriate.

Quantitative and qualitative data were analyzed separately and integrated during the interpretation of the findings, in accordance with the convergent mixed methods design. The quantitative questionnaire data were used to identify general patterns in participants’ responses regarding home monitoring, while the qualitative data from open-text responses and interviews were used to provide context and explain the reasons underlying these response patterns.

## Results

### Participant Characteristics

Of the 131 parent-child dyads who provided informed consent, 113 parent-child dyads (Juliana Children’s Hospital: n=86, 76.1%; Sophia Children’s Hospital: n=27, 23.9%) completed the questionnaire. One parent-child dyad was excluded from the analysis because the caregiver questionnaire was incomplete. Seventeen dyads did not return the questionnaire. These children were all diagnosed with asthma, had a median age of 13.0 (IQR 10-15) years, and had been approached during an outpatient clinic visit. No further clinical information or information on the reasons for noncompletion was available. Of the 113 children with completed questionnaire data, 54.9% (n=62) were diagnosed with asthma, and 45.1% (n=51) were diagnosed with CF. A total of 9 children (n=6 with asthma and n=3 with CF) did not complete the pediatric part of the questionnaire due to an inability to understand the questions. The mean pediatric age was 10.5 (SD 3.2) years in the asthma group and 12.7 (SD 3.6) years in the CF group. A total of 23 (20.4%) caregivers described the disease burden of their child as very or somewhat heavy.

Of the 113 caregivers, 46 (40.7%) reported lung complaints as the most burdensome aspect of their child’s disease, while 39 (34.5%) caregivers indicated that daily medication caused the greatest disease burden. Caregivers of children with asthma more frequently attributed the disease burden to lung complaints than did caregivers of children with CF (n=38, 61.3% vs n=8, 15.7%; *χ*^2^_1_=22.3; *P*<.001). In contrast, caregivers of children with CF more often identified daily medication use as the main contributor to disease burden than caregivers of children with asthma (n=24, 47.1% vs n=14, 22.6%; *χ*^2^_1_=6.5; *P*=.01). Additional participant characteristics are presented in Table 1.

**Table 1:** Participant characteristics (N=113).

Characteristics			Total participants		Participants with asthma (n=62)		Participants with cystic fibrosis (n=51)
Age (years), median (IQR)			11 (8.0-14.0)		10 (8.0-12.8)		13 (10.0-16.0)
Sex: male n (%)			62 (54.8)		40 (64.5)		22 (43.1)
**Distance to hospital (km), n (%)**							
	<5	43 (38.1)		34 (54.8)		9 (17.6)	
	6-20	42 (37.2)		24 (38.7)		18 (35.2)	
	>20	28 (24.8)		4 (6.5)		24 (47.1)	
**Caregiver-reported disease burden, n (%)**							
	Heavy	23 (20.4)		15 (24.2)		8 (15.7)	
	Average	36 (31.9)		16 (25.8)		20 (39.2)	
	Light	54 (47.8)		31 (50)		23 (45.1)	
**Hospital visits in the previous year**							
	Outpatient clinic visits, median (IQR)	4.0 (2.0-4.0)		3.0 (2.0-4.0)		4.0 (4.0-4.0)	
	≥1 emergency department visit, n (%)	18 (15.9)		15 (24.2)		3 (5.9)	
	≥1 hospital admission, n (%)	12 (10.6)		7 (11.3)		5 (9.8)	
**Medication, n (%)**							
	Short-acting β-agonist	51 (45.1)		46 (74.2)		5 (9.8)	
	Long-acting β-agonist	29 (25.7)		23 (37.1)		6 (11.8)	
	Inhaled corticosteroid	48 (42.5)		48 (77.4)		0 (0)	
	Leukotriene receptor antagonist	4 (3.5)		4 (6.5)		0 (0)	
	Biologic	4 (3.5)		4 (6.5)		0 (0)	
	Cystic fibrosis transmembrane conductance regulator modulator	48 (42.5)		0 (0)		48 (94.1)	
**Home monitoring, n (%)**							
	Currently using	24 (21.2)		13 (21)		11 (21.6)	
	Used previously	42 (37.2)		18 (29)		24 (47.1)	
**Device used, n (%)**							
	Spirometer	22 (19.5)		2 (3.2)		20 (39.2)	
	Oxygen level measurement	17 (15)		13 (21)		4 (7.8)	

### Home Monitoring Experience

A total of 42 (37.2%) caregivers reported that their child had used home monitoring previously (n=18, 29% caregivers of children with asthma vs n=24, 47.1% caregivers of children with CF; *χ*^2^_1_=3.2; *P*=.08). Among children with CF, the most commonly used device was a handheld spirometer, which was reported for 20 (39.2%) children. Among children with asthma, pulse oximeters were most commonly reported (n=13, 21%). According to caregivers, most children (n=30, 71.4%) primarily used home monitoring devices during symptomatic episodes rather than incorporating them into a routine for regular monitoring. Caregivers stated that reasons for discontinuing home monitoring included unreliable measurements, perceived lack of necessity, and the added uncertainty it caused. One caregiver of a child with CF stated, “it was useful in the past, it is not necessary anymore with the new medication,” indicating the availability of newer CF transmembrane conductance regulator (CFTR)–modulating therapies.

### Willingness to Use Home Monitoring

Most caregivers (98/113, 86.7%) expressed willingness to use home monitoring. About 77% (n=87) of caregivers would use home monitoring if it resulted in fewer outpatient visits. Of the 113 caregivers, 99 (87.6%) would use home monitoring if it led to fewer admissions, while 96.5% (n=109) would use it if it led to less lung damage. Summarized responses are displayed in Table 2. There were no associations between the age of the participants, diagnosis, use of home monitoring in the past, or perceived disease severity and the willingness of caregivers to use home monitoring (Multimedia Appendix 3).

Of all children, 74% (77/104) indicated willingness to use home monitoring in general. Children with asthma were significantly more willing (Likert scale score: median 2.0, IQR 1.0-2.0) than those with CF (Likert scale score: median 2.0, IQR 2.0-3.0; Mann-Whitney *U* test, *P*<.001). Age did not influence children’s willingness to use home monitoring (Multimedia Appendix 3).

**Table 2:** Willingness to use home monitoring.

Willingness to use home monitoring		Participants with asthma, n (%)	Participants with cystic fibrosis, n (%)	*P* value
**Caregivers’ perspective^a^**				
	If it was available	54 (87.1)	44 (86.3)	.99
	If it resulted in fewer outpatient visits	48 (77.4)	39 (76.5)	.99
	If it resulted in a telephonic consultation instead of an outpatient visit	50 (80.6)	36 (70.6)	.30
	If it led to fewer hospital admissions	55 (88.7)	44 (86.3)	.92
	If it led to a decrease in long-term lung damage	60 (96.8)	49 (96.1)	.99
**Children’s perspective^b^**				
	If it was available	47 (83.9)	30 (62.5)	<.001^c^
	If it resulted in fewer outpatient visits	45 (80.4)	36 (75)	.36
	If it led to fewer hospital admissions	54 (96.4)	46 (95.8)	.99

^a^Caregiver responses were based on 62 participants with asthma and 51 participants with cystic fibrosis. The questions required yes or no responses. Differences were assessed using the chi-square test.

^b^Child responses were based on 56 participants with asthma and 48 participants with cystic fibrosis. The questions used a 5-point Likert scale, and participants were considered willing if they selected “I would like to use” or “I would be fine to use.” Differences were assessed using the Mann-Whitney *U* test.

^c^*P*<.05 was considered statistically significant.

### Home Monitoring Tools

Figures 1A and 1B present caregivers’ and children’s willingness to use different home monitoring devices, respectively. Overall, caregivers’ willingness was highest for air quality meters (91/113, 80.5%), followed by daytime smartwatches (86/113, 76.1%), nighttime smartwatches (83/113, 73.5%), cough monitors (74/113, 65.5%), symptom questionnaires (72/113, 63.7%), and medication reminders (59/113, 52.2%). A total of 61 (98.4%) caregivers of the children with asthma would use an additional meter on the inhaler. Overall, children showed the greatest willingness to use a smartwatch during the daytime (87/104, 83.6%), whereas symptom questionnaires were the least preferred option (55/104, 52.8%). Acceptance of the other monitoring devices included a nighttime smartwatch (76/104, 73.1%), a spirometer (71/104, 68.2%), a cough monitor (68/104, 65.4%), an air quality meter (66/104, 63.5%), and medication reminders (65/104, 62.5%). The additional meter on the inhaler was acceptable for 78.6% (44/56) of children with asthma.

Among both children and caregivers, all devices were significantly more often preferred by participants with asthma, except for symptom questionnaires in the pediatric population and medication reminders in the caregiver population (Multimedia Appendix 4). Previous use of home spirometry in the pediatric population was not associated with willingness to use a spirometer in this questionnaire (Likert scale score median 2.0, IQR 2.0-3.0 vs median 2.0, IQR 1.0-3.0; Mann-Whitney *U* test, *P*=.50).

To summarize caregivers’ and children’s overall attitudes across the different home monitoring modalities, a cumulative device score was calculated and displayed in Figures 2A (asthma) and 2B (CF). Higher scores represent greater overall willingness to use the proposed home monitoring modalities. A higher subjective disease burden was significantly associated with a higher cumulative device score in both asthma caregivers (Spearman rank correlation coefficient ρ=0.29; *P*=.02) and CF caregivers (Spearman rank correlation coefficient ρ=0.40; *P*=.003).

**Figure 1:**
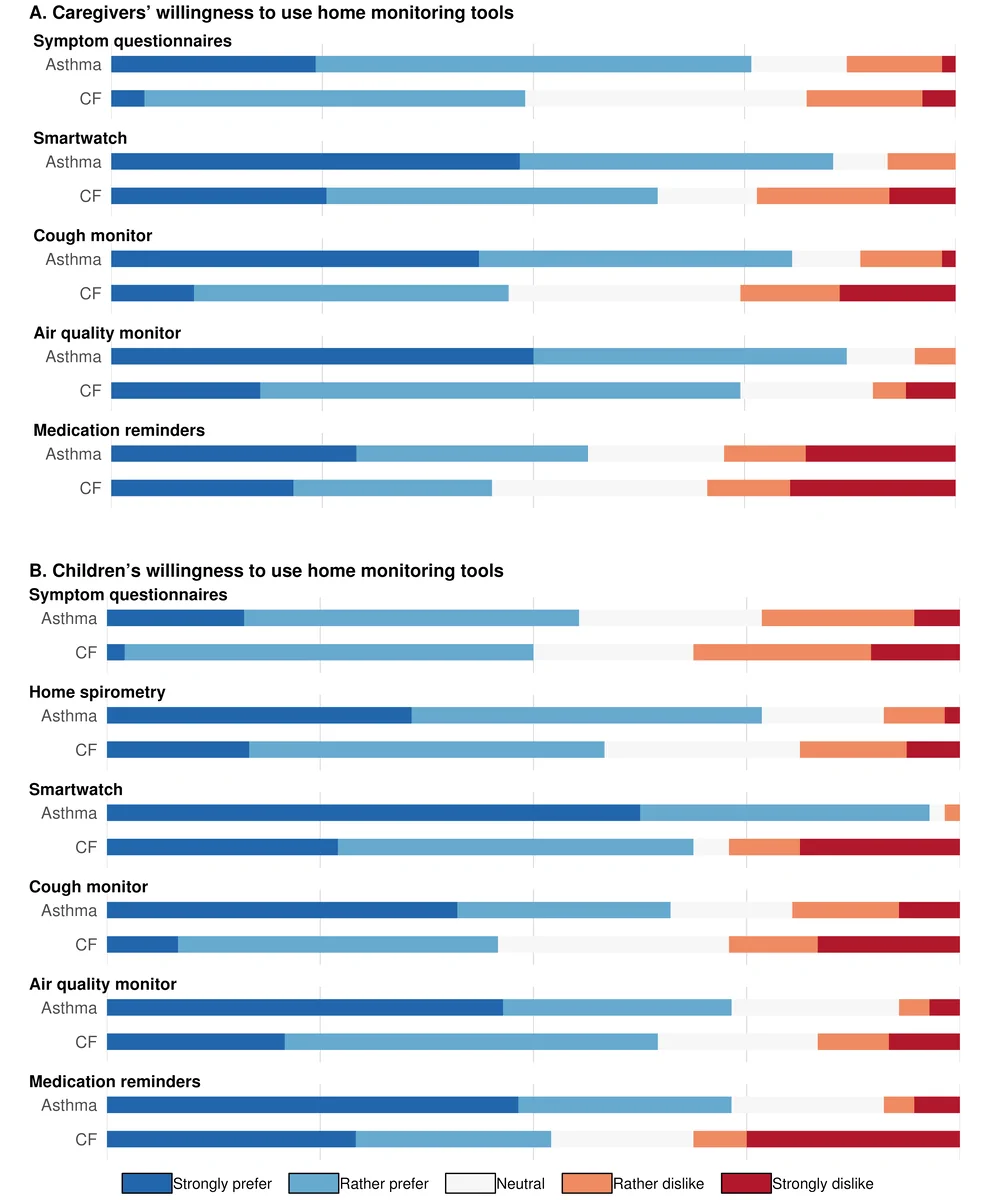
Willingness to use home monitoring tools. (A) Caregivers’ willingness to use different home monitoring tools for children with asthma and cystic fibrosis (CF) and (B) children’s willingness to use different home monitoring tools.

**Figure 2:**
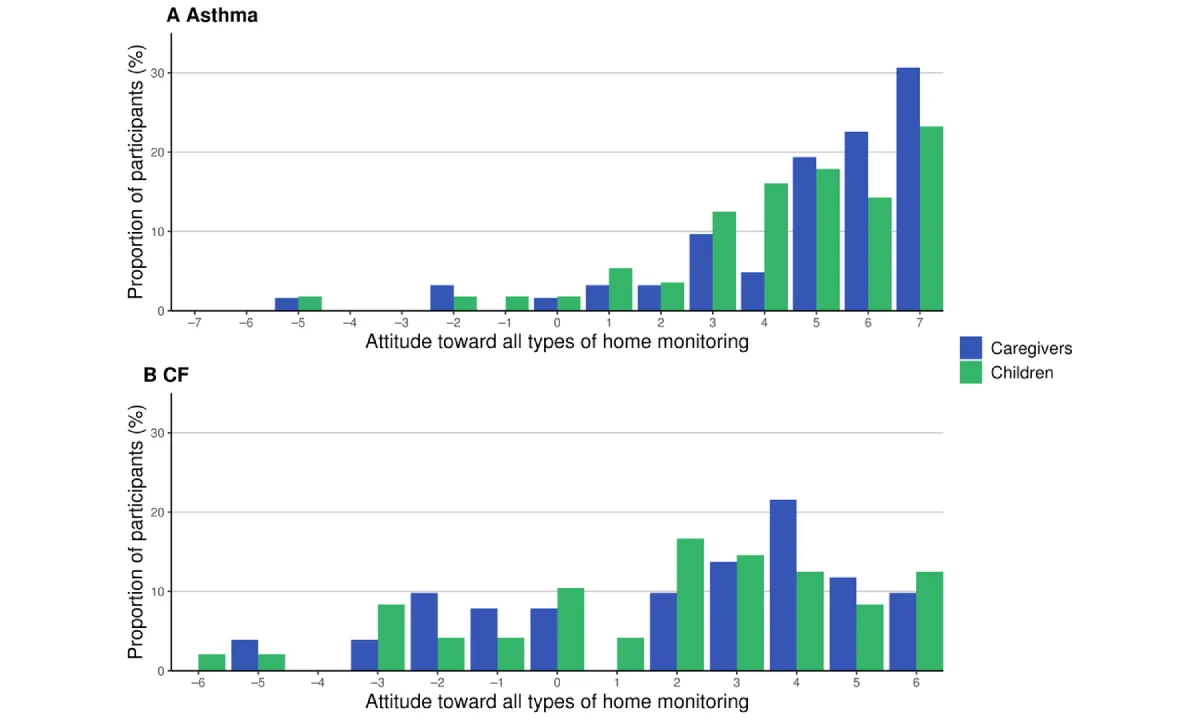
Distribution of the cumulative device willingness score among caregivers and children with (A) asthma and (B) cystic fibrosis (CF). For each applicable home monitoring modality, willingness to use the modality was scored as +1, unwillingness as –1, and a neutral response or no opinion as 0. The maximum possible cumulative score was 7 in the children with asthma and 6 in the children with CF.

### Symptom Interpretation

Symptom recognition was generally comparable between the asthma and CF groups (Multimedia Appendix 5). A total of 69 (61.1%) caregivers reported an accurate assessment of disease symptoms. About 42.5% (48/113) of caregivers reported that when they first noticed symptoms, they believed those symptoms had already been present for >1 day. Caregivers who expressed difficulty with symptom interpretation were significantly more motivated to use home monitoring in general (Spearman rank correlation coefficient ρ=–0.19; *P*=.04) and had a higher cumulative device score (Spearman rank correlation coefficient ρ=–0.272; *P*=.004). This correlation was not significant in children who expressed difficulty with symptom interpretation (Spearman rank correlation coefficient ρ=–0.18; *P*=.07). Multimedia Appendix 5 shows the ability of caregivers and children to notice disease-specific complaints.

### Data Interpretation

Of the 113 caregivers, 82 (72.6%) would only use home monitoring if insight into the results was provided (n=42, 67.7% caregivers of children with asthma vs n=40, 78.4% caregivers of children with CF; *χ*^2^=1.1; *P*=.30), while 72.6% (n=82) would only use home monitoring if a physician reviewed the results regularly (n=46, 74.2% caregivers of children with asthma vs n=36, 70.6% caregivers of children with CF; *χ*^2^=0.05; *P*=.83). Only 38.1% (n=43) of caregivers would follow algorithmically generated treatment advice before discussing the results with their physicians, although the asthma cohort was significantly more willing to follow algorithmically generated treatment advice without consulting a medical specialist than caregivers in the CF cohort (n=30, 48.4% vs n=13, 25.5%; *χ*^2^_2_=6.4; *P*=.04).

### Barriers and Facilitators

In total, 14 children (n=8 with asthma and n=6 with CF) and their caregivers also took part in an interview to elaborate on their answers. A total of 88 (77.8%) caregivers answered the open-ended question about expected barriers, and 79 (69.9%) caregivers answered the question about expected facilitators.

Responses were generally similar between the asthma and CF groups. Content analysis of barriers and facilitators identified the following themes: time commitment, ease of use, safety, and motivation, as displayed in Figure 3. The main barrier was the perceived time burden of home monitoring (n=23). Other caregivers highlighted the importance of user-friendly, compact devices that minimally disrupt the child’s daily life (n=19). The child’s motivation (n=6) was another possible barrier to the use of home monitoring. Adoption of devices would be enhanced by clear instructions before use and education on the interpretation of the results (n=15). Caregivers also expressed concerns about device safety, privacy, and electromagnetic exposure (n=8), emphasizing the importance of trust in the equipment. Finally, worries about reduced patient-physician interaction (n=6) were accentuated by the desire for continued contact with physicians or specialized nurses to interpret results and maintain a human-centered approach to care (n=6). In addition, several caregivers (n=12) indicated that home monitoring could enhance their understanding of their child’s illness and help them anticipate clinical deterioration.

**Figure 3:**
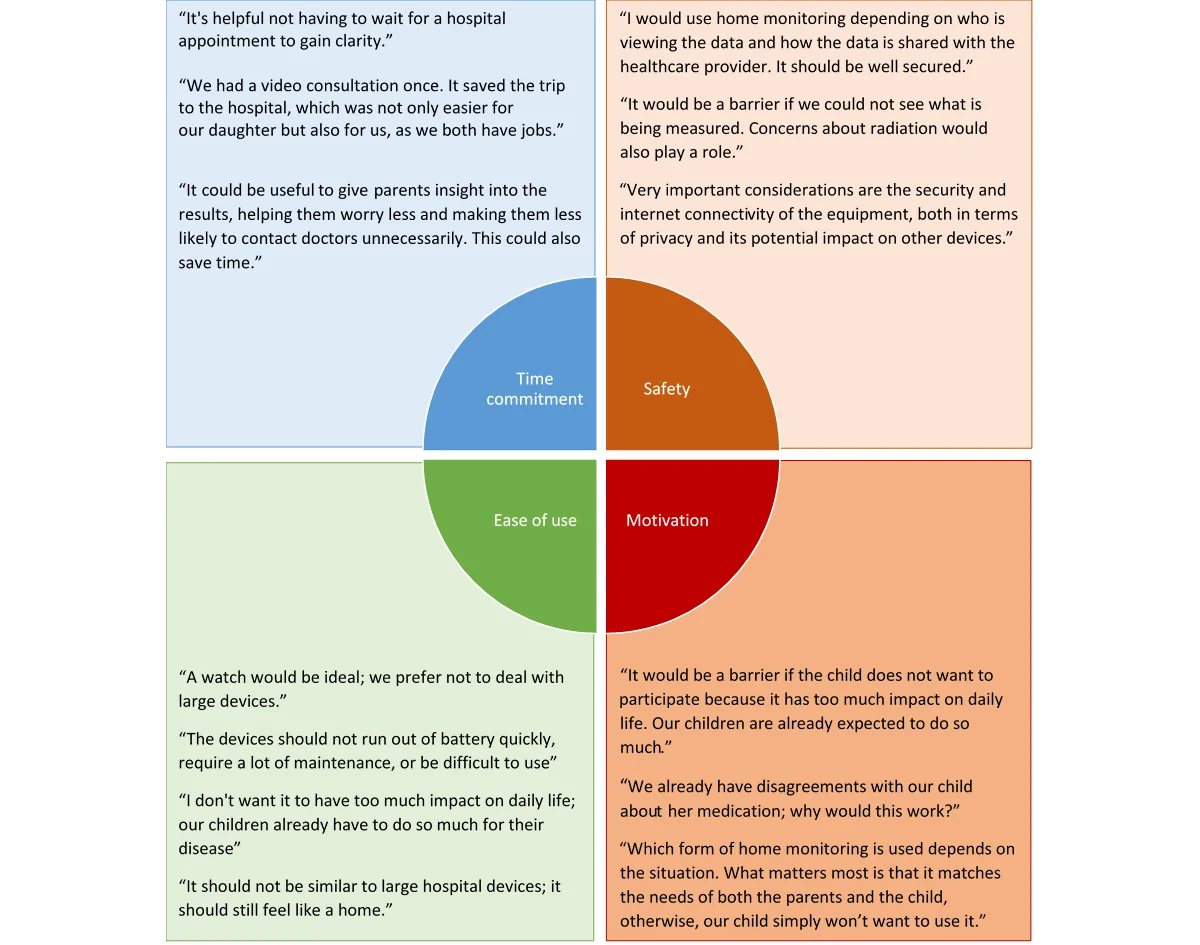
Content analysis of barriers and facilitators of home monitoring. Four main themes—time commitment, safety, ease of use, and motivation—are illustrated with representative participant quotes.

## Discussion

### Principal Findings

Home monitoring may support early detection of disease activity in pediatric lung disease, but its implementation in clinical practice remains limited [20-23]. This study explored perspectives, barriers, and facilitators related to home monitoring in pediatric pulmonology and showed that 86.7% (98/113) of caregivers and 74% (77/104) of children expressed a general willingness to use home monitoring. Several important factors were identified that should be addressed in future research, development, and implementation of home monitoring.

Caregivers were particularly motivated to use home monitoring if it could reduce long-term lung damage or lead to fewer hospital admissions. In addition, access to data and physician involvement were shown to be important factors for caregivers. Associations with a positive attitude toward multiple home monitoring modalities were found for caregivers of children with a higher disease burden and lower proficiency in symptom recognition, while age did not appear to be a significant factor. These results suggest that researchers should focus on gathering evidence that consistently shows whether home monitoring systems may improve long-term outcomes. However, previous studies on this subject have demonstrated that home monitoring did not achieve better long-term outcomes [24-27]. Additionally, it appears that children and caregivers value having access to their own health data and guidance in interpreting these results, as this may support symptom recognition and enhance their sense of control. This observation is in line with previous literature showing that access to personal health data can improve illness perception [28-31].

The qualitative component of this study provides context for the generally high willingness observed in the quantitative results by identifying important barriers to the implementation of home monitoring in practice. In line with previous literature, time commitment and sustained motivation were key barriers to implementation [32,33], as well as ease of use. Therefore, future research should focus on passive monitoring with “dummy proof” devices and minimize active measurements as much as possible. Caregivers also stressed the need for continued physician involvement in this section of the questionnaire. These barriers may help explain the gap between the apparent willingness to use home monitoring and its limited uptake in current clinical practice [34,35]. Home spirometry is a well-studied example: although widely investigated [36,37], long-term use is hampered by declining measurement quality [38] and reduced motivation over time [10].

Several notable differences between the asthma and CF groups were found in this study. These differences should be interpreted with caution, as the groups also differed in several participant characteristics, including age, sex, distance from the hospital, and previous experience with home monitoring, which may have contributed to the observed differences. In both children with asthma and their caregivers, home monitoring of pulmonary symptoms seemed to align well with patient needs, while caregivers and children in the CF group showed less interest in monitoring focused specifically on pulmonary symptoms. This may partly reflect the introduction of modern CFTR modulators, which have substantially improved lung function and symptom control in people with CF [39]. This may also be explained by the more variable and episodic nature of symptoms in asthma, which can develop rapidly, whereas exacerbations in CF are often preceded by a more gradual onset of symptoms.

A strength of our study is that, to our knowledge, it is one of the largest cohorts to examine the perspectives of children and caregivers on home monitoring. Including both perspectives provides a broader understanding of preferences and expectations. In addition, participants represented a range of ages and disease severities, improving the representativeness of the sample. Finally, the combination of quantitative and qualitative data provided a deeper understanding of motivations, concerns, and needs relevant to implementation in pediatric pulmonary care. The main limitation of this study is that it relied on self-reported data via a questionnaire that was not previously validated in other studies, which may have induced recall bias or social desirability bias. However, the questionnaire was developed through several feedback loops to improve face validity, clarity, and relevance for the target population. The sample in this study was drawn from 2 large urban hospitals in the Netherlands, which may limit the generalizability of the findings to broader, more diverse, or rural populations. Moreover, 51 parent-child dyads were included in the CF group rather than the planned 60, as the rarity of the disease limited the achievable sample size within the recruitment period. The relatively small sample size, particularly within subgroup analyses, may have limited the ability to detect significant associations. Despite not reaching the predefined target, the collected data were sufficient to provide a comprehensive overview. No correction for multiple testing was applied, as the analyses were exploratory in nature. In addition, as interviews were conducted in a subset of participants, selection bias cannot be excluded for this analysis, and the qualitative findings may not capture the full range of perspectives within the study population. Further research with a more diverse sample and a longitudinal design may lead to a better understanding of the implementation of home monitoring programs for children with asthma and children with CF. Finally, future work with a quantitative or qualitative approach should gather insights from children with other lung diseases.

### Conclusions

Interest in home monitoring is high among children with asthma and CF and their caregivers, particularly in families with poorer symptom recognition. To translate this interest into clinical practice, home monitoring strategies should demonstrate clear clinical benefit, minimize patient burden through user-friendly or passive systems, and be integrated into routine care with active physician involvement.

## Appendix

Multimedia Appendix 1 GRAMMS (Good Reporting of a Mixed Methods Study) checklist.

Multimedia Appendix 2 Questionnaire.

Multimedia Appendix 3 Statistical analysis and variables influencing willingness to use home monitoring across subgroups.

Multimedia Appendix 4 Median Likert scale score for different types of home monitoring tools for asthma and cystic fibrosis.

Multimedia Appendix 5 Ability of caregivers and children to notice disease-specific symptoms.

## Abbreviations

CF: cystic fibrosis
CFTR: cystic fibrosis transmembrane conductance regulator
GINA: Global Initiative for Asthma
GRAMMS: Good Reporting of a Mixed Methods Study
WMO: Wet Medisch-Wetenschappelijk Onderzoek Met Mensen

## Data Availability

All data presented in this manuscript are available from the corresponding author upon reasonable request.
