# Supplementary material for: Exploring Perspectives and Barriers in Implementing Home Monitoring for Pediatric Pulmonary Diseases: Mixed Methods Study
Source: JMIR Pediatr Parent. 2026 Oct 20;9:e101452. doi: 10.2196/101452 (PMC13732169; doi:10.2196/101452)
Supplement: Supplementary file 1 [file pediatrics_v9i1e101452_app2.pdf]

Papieren vragenlijst over de cognities van ouders en kinderen over thuis monitoren van astma en taaislijmziekte.

Geachte ouder/verzorger,

Hartelijk dank voor deelname aan dit onderzoek. Wij waarderen uw deelname zeer, en kijken uit naar uw ideeën over het gebruik van apparatuur voor het thuismonitoren van de ziekte van uw kind. De vragen in deze vragenlijst zijn gesteld vanuit het perspectief van de ouder. Wij willen u vragen om de vragenlijst samen met uw kind in te vullen, zodat de uitkomst van dit onderzoek zowel de gedachten van u en uw kind behelzen. Als de mening van u en uw kind verschillen, willen we u vragen dit aan te geven bij de toelichting van uw antwoord op de vraag.
Het laatste deel van de vragenlijst zal (indien mogelijk) een vragenlijst alleen voor het kind zijn.

Vriendelijke groeten, het onderzoeksteam

Algemeen

1. Mijn kind is gediagnosticeerd met
   - ☐ Astma, op de leeftijd van _____ jaar
   - ☐ Cystische fibrose (taaislijmziekte), op de leeftijd van _____ jaar

2. Mijn kind is tevens bekend met de volgende aandoeningen:

____
____

3. Mijn kind heeft klachten van de longen sinds de leeftijd van _____ jaar

4. De leeftijd van mijn kind is _____ jaar.

5. Het geslacht van mijn kind is:
   - ☐ Jongen
   - ☐ Meisje

6. Wij wonen ... km van het ziekenhuis.
   - ☐ <2 km
   - ☐ 2-5 km
   - ☐ 5-10 km
   - ☐ 10-20 km
   - ☐ 20-50 km
   - ☐ >50 km
7. Wij komen ... naar het ziekenhuis:
   - ☐ Lopend
   - ☐ Met de fiets
   - ☐ Met de auto
   - ☐ Met de scooter/motor
   - ☐ Met het openbaar vervoer

8. Wij doen hier ______ minuten over

9. Mijn kind is in de laatste 12 maanden ______ keer op de poli geweest voor controle.

10. Mijn kind is in de laatste 12 maanden ______ keer op de SEH geweest vanwege een longaanval of verergering van de longziekte

11. Mijn kind is in de laatste 12 maanden ______ keer opgenomen vanwege een longaanval of verergering van de longziekte.

12. Mijn kind is in de laatste 24 maanden ______ keer opgenomen vanwege een longaanval of verergering van de longziekte.

13. Mijn kind gebruikt op dit moment de volgende medicatie (patiënten met astma):
- ☐ Kortwerkende bronchusverwijders (Salbutamol)
  - o Hoeveel keer per week? _____keer
- ☐ Inhalatie corticosteroïden
  - o (Zoals pulmicort, qvar, flixotide, budesonide, beclometason, fluticasone, ciclesonide)
- ☐ Langwerkende bronchusverwijders (zoals formoterol, salmeterol)
- ☐ Montelukast
- ☐ Immuuntherapie (mepolizumab, omalizumab, dupilumab)
- ☐ Anders, namelijk: ____

14. Mijn kind gebruikt op dit moment de volgende medicatie (patiënten met CF):
- ☐ Kortwerkende bronchusverwijder(Salbutamol)
- ☐ Langwerkende bronchusverwijder (Seretide of Foster)
- ☐ Vernevelaar (Pulmozyme , Hypertoon zoutverneveling)
- ☐ CF modulator (Kalydeco, Orkambi, Symkevi, Kaftrio)
- ☐ Antibiotica (Cotrimoxazol of Azitromycine)
- ☐ Neusspray (Flixonase)
- ☐ Kreon
- ☐ Vitamine A, D, E, K
- ☐ Anders, namelijk: ____

15. Ik vind de ziektelast voor mijn kind
- ☐ Erg licht.
- ☐ Licht
- ☐ Gemiddeld.
- ☐ Zwaar
- ☐ Erg zwaar.

16. Het grootste van de ziektelast van mijn kind wordt bepaald door
- ☐ De klachten van de longen
- ☐ Het dagelijks gebruik van medicatie
- ☐ De polibezoeken
- ☐ De opnames

☐ Anders, namelijk:
____

17. Ik vind het lastig om in te schatten of mijn kind last heeft van klachten die bij de ziekte horen:

☐ Helemaal mee eens
☐ Mee eens
☐ Neutraal
☐ Niet mee eens
☐ Helemaal niet mee eens

Toelichting:
____
____

18. Ik vind het lastig om in te schatten of mijn kind last heeft van benauwdheid.

☐ Helemaal mee eens
☐ Mee eens
☐ Neutraal
☐ Niet mee eens
☐ Helemaal niet mee eens

Toelichting:
____
____

19. Het verminderen van het aantal polibezoeken zou de ziektelast van mijn kind doen verminderen.

☐ Helemaal mee eens
☐ Mee eens
☐ Neutraal
☐ Niet mee eens
☐ Helemaal niet mee eens

Toelichting:
____
____

20. Ik vind dat een bezoek een de poli meerwaarde heeft vanwege:

☐ Het gesprek met de arts
☐ Het lichamelijk onderzoek
☐ Anders, namelijk: ____

21. Ik merk de volgende symptomen aan mijn kind als hij/zij een longaanval heeft:

____
____
____

22. Op het moment dat ik merk dat mijn kind klachten heeft, zijn deze klachten achteraf gezien al meer dan een dag aanwezig.

☐ Helemaal mee eens
☐ Mee eens
☐ Neutraal
☐ Niet mee eens
☐ Helemaal niet mee eens

Toelichting:

____

____

Eerdere ervaring met thuismonitoring apparatuur

23. Heeft u in het verleden nagedacht over het gebruik van apparatuur om thuis de ziekte van uw kind te monitoren?
   - ☐ Ja, ik dacht toen over____
   - ☐ Nee

24. Heeft u in het verleden met de behandelend arts van uw kind gesproken over apparatuur om thuis de ziekte van uw kind te monitoren?
   - ☐ Ja, we spraken toen over ____
   - ☐ Nee

25. Heeft u in het verleden apparatuur gebruikt om thuis de ziekte van uw kind te monitoren?
   - ☐ Ja
     - ➔ Ik heb het volgende gebruikt:____
   - ☐ Nee

26. Maakt u momenteel gebruik van thuismonitoringssystemen?
   - ☐ Ja (ga naar vraag 27)
   - ☐ Nee (ga naar vraag 31)

27. Welke thuismonitoring gebruikt u?____

28. Hoe vaak?
   - ☐ Altijd
   - ☐ Alleen overdag
   - ☐ Alleen ’s nachts
   - ☐ Meerdere keren per dag
   - ☐ Dagelijks
   - ☐ Wekelijks
   - ☐ Maandelijks
   - ☐ Alleen als mijn kind klachten heeft
   - ☐ Nooit

29. Hoe vindt u het om hier gebruik van te maken?

   ____

   ____

30. Heeft u het idee dat het gebruik van de thuismonitoring u voordelen geeft? Zo ja welke?

   ____

   ____

   ____

   De volgende twee vragen hoeft u niet te maken, u mag nu door naar vraag 33.

31. Heeft u de optie wel gekregen?
   - ☐ Ja

☐ Nee

32. Waarom heeft u het toen niet gedaan?

____

____

33. Denkt u dat thuismonitoring door middel van digitale apparatuur (smartwatch, longfunctiemeter, symptoomdagboek) een goede inschatting kan geven over de ziektelast van uw kind?

☐ Ja
☐ Nee
☐ Misschien.

Toelichting:

____

____

____

34. Zou u het gebruik van apparatuur overwegen om de ziekte van uw kind te monitoren?

☐ Ja
☐ Nee

Toelichting:

____

____

____

35. Zou u het gebruik van apparatuur overwegen om de ziekte van uw kind te monitoren, als dit betekent dat uw kind minder vaak op de poli gezien hoeft te worden?

☐ Ja
☐ Nee

Toelichting:

____

____

____

36. Zou u het gebruik van apparatuur overwegen om de ziekte van uw kind te monitoren, als dit betekent dat uw kind minder vaak op de poli gezien hoeft te worden, maar dat in plaats hiervan een telefonische afspraak wordt ingepland om de meetwaarden van de apparatuur te bespreken?

☐ Ja
☐ Nee

Toelichting:

____

____

____

37. Zou u het gebruik van apparatuur overwegen om de ziekte van uw kind te monitoren, als uit onderzoek blijkt dat uw kind minder vaak hoeft te worden opgenomen in het ziekenhuis?

☐ Ja
☐ Nee

Toelichting:

____
____
____

38. Zou u het gebruik van apparatuur overwegen om de ziekte van uw kind te monitoren, als uit onderzoek blijkt dat de longen van uw kind hierdoor minder schade oplopen op de lange termijn?
    - ☐ Ja
    - ☐ Nee

    Toelichting:

    ____
    ____
    ____

<u>Specifieke apparatuur</u>

Een smartwatch zou dingen kunnen meten als de hartslag, lichamelijke activiteit en slaap van het kind om op die manier mogelijk een inschatting te kunnen maken van de status van de ziekte en het mogelijk aankomen van een longaanval.

39. Wat zou u vinden van het overdag dragen van een smartwatch door uw kind, om de ziekte van uw kind te monitoren?
    - ☐ Ik zou dit fijn vinden.
    - ☐ Ik zou dit prima vinden.
    - ☐ Ik heb hier geen mening over.
    - ☐ Ik zou dit vervelend vinden.
    - ☐ Ik zou dit heel vervelend vinden.

    Toelichting:

    ____
    ____

40. Wat zou u vinden van het ‘s nachts dragen van een smartwatch door uw kind, om de ziekte van uw kind te monitoren?
    - ☐ Ik zou dit fijn vinden.
    - ☐ Ik zou dit prima vinden.
    - ☐ Ik heb hier geen mening over.
    - ☐ Ik zou dit vervelend vinden.
    - ☐ Ik zou dit heel vervelend vinden.

    Toelichting:

    ____
    ____

Een longfunctiemeter kan iets zeggen over de longinhoud en op die manier mogelijk een inschatting te kunnen maken van de status van de ziekte en het mogelijk aankomen van een longaanval.

41. Hoe vaak zou u een longfunctiemeter maximaal willen gebruiken:
    - ☐ Dagelijks
    - ☐ Wekelijks

☐ Maandelijks
☐ Alleen als mijn kind klachten heeft

Toelichting:
____
____

42. Wat zou u vinden van het gebruik van een extra meter op de puffer(s) van uw kind om de ziekte van uw kind te monitoren?
   ☐ Ik zou dit fijn vinden.
   ☐ Ik zou dit prima vinden.
   ☐ Ik heb hier geen mening over.
   ☐ Ik zou dit vervelend vinden.
   ☐ Ik zou dit heel vervelend vinden.

   Toelichting:
   ____
   ____

Een hoestdetector kan door middel van het meten hoe vaak een kind hoest mogelijk een inschatting te kunnen maken van de status van de ziekte en het mogelijk aankomen van een longaanval.

43. Wat zou u vinden van het plaatsen van een apparaat naast het bed van uw kind om te meten hoe vaak uw kind hoest gedurende de nacht?
   ☐ Ik zou dit fijn vinden.
   ☐ Ik zou dit prima vinden.
   ☐ Ik heb hier geen mening over.
   ☐ Ik zou dit vervelend vinden.
   ☐ Ik zou dit heel vervelend vinden.

   Toelichting:
   ____
   ____

44. Wat zou u vinden van het plaatsen van een apparaat naast het bed van uw kind om de luchtkwaliteit te meten?
   ☐ Ik zou dit fijn vinden.
   ☐ Ik zou dit prima vinden.
   ☐ Ik heb hier geen mening over.
   ☐ Ik zou dit vervelend vinden.
   ☐ Ik zou dit heel vervelend vinden.

   Toelichting:
   ____
   ____

Een vragenlijst kan met behulp van symptoomscores mogelijk een inschatting te kunnen maken van de status van de ziekte en het mogelijk aankomen van een longaanval.

45. Wat zou u vinden van het invullen van een vragenlijst om de ziekte van uw kind te volgen?
   ☐ Ik zou dit fijn vinden.
   ☐ Ik zou dit prima vinden.
   ☐ Ik heb hier geen mening over.
   ☐ Ik zou dit vervelend vinden.

☐ Ik zou dit heel vervelend vinden.

Toelichting:

____
____

46. Hoe vaak zou u die vragenlijst maximaal willen invullen?
    - ☐ Dagelijks
    - ☐ Wekelijks
    - ☐ Maandelijks
    - ☐ Alleen als mijn kind klachten heeft

    Toelichting:

    ____
    ____

47. Uit hoeveel vragen zou die vragenlijst wat u betreft mogen bestaan? ________vragen

48. Hoeveel minuten per week zou u maximaal kwijt willen zijn aan het gebruik van de apparatuur voor thuismonitoring? ________ minuten

48. Een herinnering of melding via een app op een telefoon zou helpen bij het dagelijks innemen van de medicatie.
    - ☐ Helemaal mee eens
    - ☐ Mee eens
    - ☐ Neutraal
    - ☐ Niet mee eens
    - ☐ Helemaal niet mee eens

    Toelichting:

    ____
    ____

Toekomst

49. Ik zou het gebruik van deze apparatuur **alleen** overwegen als ik zelf de gegevens kan inzien.
    - ☐ Eens
    - ☐ Oneens
    - ☐ Geen mening

    Toelichting:

    ____
    ____

50. Ik zou het gebruik van deze apparatuur **alleen** overwegen als ik weet dat een arts regelmatig naar de gegevens kijkt en deze interpreteert.
    - ☐ Eens
    - ☐ Oneens
    - ☐ Geen mening

    Toelichting:

____
____

51. Wie vindt u dat de meetgegevens het beste zou kunnen interpreteren?
    - ☐ De ouders, eventueel samen met hun kind
    - ☐ De arts
    - ☐ Een computer, op basis van een algoritme
    - ☐ Geen mening

    Toelichting:

    ____
    ____

52. Als een app de meetgegevens van meerdere apparaten door middel van een algoritme zou interpreteren en daarna zelfstandig een behandeladvies geeft, zou ik dat opvolgen.
    - ☐ Eens
    - ☐ Oneens
    - ☐ Geen mening

    Toelichting:

    ____
    ____

53. Als een app de meetgegevens van meerdere apparaten door middel van een algoritme zou interpreteren en daarna zelfstandig een behandeladvies geeft, zou ik dat eerst met een arts willen bespreken.
    - ☐ Eens
    - ☐ Oneens
    - ☐ Geen mening

    Toelichting:

    ____
    ____

54. Als ik de meetgegevens interpreteer en zie dat er achteruitgang te zien is bij mijn kind, zou ik het volgende doen:

    ____
    ____
    ____

Barrières en helpende factoren

55. Wat voor barrières kunnen u en uw kind indenken die jullie zouden belemmeren bij het gebruik een thuismonitoring systeem?

____
____
____
____

56. Wat zou jullie helpen bij het gebruik van een thuismonitoring systeem bij uw kind?

____
____
____
____

Dit was de laatste vraag van de vragenlijst voor ouders. Hartelijk dank voor het invullen!

Vragen voor het kind

Dit is een vragenlijst met allemaal vragen over jouw ziekte. Wij willen graag meer weten over jouw ziekte en hoe je over dingen nadenkt. Probeer deze vragen alsjeblieft zo goed als mogelijk te beantwoorden. Als je niet goed weet wat er met de vraag bedoeld wordt, is dat helemaal niet erg. Laat hem in dat geval leeg, of bespreek de vraag met jouw ouders of de onderzoeker.

1. Ik merk de volgende symptomen als ik een longaanval heb:

____
____
____
____

2. Ik vind het lastig om te merken dat ik last heb van benauwdheid.

☐ Helemaal mee eens
☐ Mee eens
☐ Neutraal
☐ Niet mee eens
☐ Helemaal niet mee eens

Toelichting:

____
____
____

3. Ik vind het leuk om naar het ziekenhuis te gaan voor controle.

☐ Helemaal mee eens
☐ Mee eens
☐ Neutraal
☐ Niet mee eens
☐ Helemaal niet mee eens

Toelichting:

____
____
____

4. Zou jij wel of niet apparaten gebruiken om hiermee te meten hoeveel last jij van je longen hebt?

☐ Ik zou dit fijn vinden.
☐ Ik zou dit prima vinden.
☐ Ik heb hier geen mening over.
☐ Ik zou dit vervelend vinden.
☐ Ik zou dit heel vervelend vinden.

Toelichting:

____
____

5. Zou je apparaten gebruiken als je hierdoor minder vaak in het ziekenhuis hoeft te komen voor controle?

☐ Ik zou dit fijn vinden.
☐ Ik zou dit prima vinden.
☐ Ik heb hier geen mening over.
☐ Ik zou dit vervelend vinden.
☐ Ik zou dit heel vervelend vinden.

Toelichting:

____
____
____

6. Zou je apparaten gebruiken als je hierdoor minder vaak in het ziekenhuis voor één of meer nachten moet blijven?

☐ Ik zou dit fijn vinden.
☐ Ik zou dit prima vinden.
☐ Ik heb hier geen mening over.
☐ Ik zou dit vervelend vinden.
☐ Ik zou dit heel vervelend vinden.

Toelichting:

____
____
____

7. Zou je een horloge willen dragen die continue meet hoe het met jou gaat?

☐ Ik zou dit fijn vinden.
☐ Ik zou dit prima vinden.
☐ Ik heb hier geen mening over.
☐ Ik zou dit vervelend vinden.
☐ Ik zou dit heel vervelend vinden.

Toelichting:

____
____
____

8. Zou je dit horloge ook ’s nachts dragen als dat gevraagd wordt?

☐ Ik zou dit fijn vinden.

- ☐ Ik zou dit prima vinden.
- ☐ Ik heb hier geen mening over.
- ☐ Ik zou dit vervelend vinden.
- ☐ Ik zou dit heel vervelend vinden.

Toelichting:

____

____

____

9. Zou je een longfunctiemeter gebruiken?
   - ☐ Ik zou dit fijn vinden.
   - ☐ Ik zou dit prima vinden.
   - ☐ Ik heb hier geen mening over.
   - ☐ Ik zou dit vervelend vinden.
   - ☐ Ik zou dit heel vervelend vinden.

   Toelichting:

   ____

   ____

   ____

10. Hoe vaak zou je maximaal een longfunctiemeter willen gebruiken?
    - ☐ Elke dag
    - ☐ Elke week
    - ☐ Elke maand
    - ☐ Alleen als ik klachten heb

11. Zou je een extra meter op jouw puffer gebruiken die meet hoe vaak je de puffer gebruikt?
    - ☐ Ik zou dit fijn vinden.
    - ☐ Ik zou dit prima vinden.
    - ☐ Ik heb hier geen mening over.
    - ☐ Ik zou dit vervelend vinden.
    - ☐ Ik zou dit heel vervelend vinden.

    Toelichting:

    ____

    ____

    ____

12. Zou je een apparaat naast jouw bed willen leggen die meet hoe vaak je hoest in de nacht?
    - ☐ Ik zou dit fijn vinden.
    - ☐ Ik zou dit prima vinden.
    - ☐ Ik heb hier geen mening over.
    - ☐ Ik zou dit vervelend vinden.
    - ☐ Ik zou dit heel vervelend vinden.

    Toelichting:

    ____

    ____

    ____

13. Zou je een apparaat naast jouw bed willen leggen die meet hoe schoon de lucht is in de kamer?
- ☐ Ik zou dit fijn vinden.
- ☐ Ik zou dit prima vinden.
- ☐ Ik heb hier geen mening over.
- ☐ Ik zou dit vervelend vinden.
- ☐ Ik zou dit heel vervelend vinden.

Toelichting:

____
____

14. Wat zou je vinden van het invullen van een vragenlijst om te volgen hoe het gaat met je ziekte?
- ☐ Ik zou dit fijn vinden.
- ☐ Ik zou dit prima vinden.
- ☐ Ik heb hier geen mening over.
- ☐ Ik zou dit vervelend vinden.
- ☐ Ik zou dit heel vervelend vinden.

Toelichting:

____
____
____

15. Hoe vaak zou je deze vragenlijst maximaal willen invullen?
- ☐ Elke dag
- ☐ Elke week
- ☐ Elke maand
- ☐ Alleen als ik klachten heb

Toelichting:

____
____
____

16. Uit hoeveel vragen zou die vragenlijst wat jou betreft mogen bestaan? _______vragen

17. Hoeveel minuten per dag zou jij maximaal kwijt willen zijn aan de thuismetingen? _______minuten

18. Een herinnering of melding via een app op een telefoon zou helpen om ervoor te zorgen dat ik mijn medicijnen elke dag inneem.
- ☐ Helemaal mee eens
- ☐ Mee eens
- ☐ Neutraal
- ☐ Niet mee eens
- ☐ Helemaal niet mee eens

Toelichting:

____
____
____

Dit was de laatste vraag. Je bent nu klaar. Dank je wel voor het invullen!
