# Supplementary material for: Exploring Perspectives and Barriers in Implementing Home Monitoring for Pediatric Pulmonary Diseases: Mixed Methods Study
Source: JMIR Pediatr Parent. 2026 Oct 20;9:e101452. doi: 10.2196/101452 (PMC13732169; doi:10.2196/101452)
Supplement: Supplementary file 2 [file pediatrics_v9i1e101452_app1.docx]

**Appendix 4 Good Reporting of A Mixed Methods Study (GRAMMS)**

| **Guideline** | **Section: page** |
| --- | --- |
| Describe the justification for using a mixed methods approach to the research question | Study design: p7 |
| Describe the design in terms of the purpose, priority and sequence of methods | Recruitment and study procedures: 7-9 |
| Describe each method in terms of sampling, data collection and analysis | Methods: p8-10 |
| Describe where integration has occurred, how it has occurred and who has participated in it | Study procedures: p8 |
| Describe any limitation of one method associated with the present of the other method | Strengths and limitations: p25-26 |
| Describe any insights gained from mixing or integrating methods | Discussion: p23-26 |

*O'Cathain A, Murphy E, Nicholl J. The quality of mixed methods studies in health services research. J Health Serv Res Policy. 2008;13(2):92-98. (13)*
