# Supplementary material for: Exploring Perspectives and Barriers in Implementing Home Monitoring for Pediatric Pulmonary Diseases: Mixed Methods Study
Source: JMIR Pediatr Parent. 2026 Oct 20;9:e101452. doi: 10.2196/101452 (PMC13732169; doi:10.2196/101452)
Supplement: Supplementary file 3 [file pediatrics_v9i1e101452_app3.docx]

Multimedia Appendix 3. Statistical analysis and variables influencing willingness to use home monitoring across subgroups.

| **Willing to use home monitoring (caregivers)** | **Yes** | **No** | **P-value**^b^ |
| --- | --- | --- | --- |
| Asthma | 54 (87.1%) | 8 (12.9%) | 1.0 |
| CF | 44 (86.3%) | 7 (13.7%) |  |
| Age < 12 years old | 51 (86.4%) | 8 (13.6%) | 1.0 |
| Age > 11 years old | 47 (87.0%) | 7 (13.0%) |  |
| Home monitoring use in the past | 37 (88.1%) | 5 (11.9%) | .97 |
| No home monitoring use in the past | 61 (85.9%) | 10 (14.1%) |  |
| **Willing to use home monitoring (pediatric)** | **Yes** | **No** | **P-value**^b^ |
| Age <12 years old (n=44)^a^ | 38 (86.4%) | 6 (13.6%) | 1.0 |
| Age >11 years old (n=46)^a^ | 39 (84.8%) | 7 (15.2%) |  |

^a^ Not all children responded to this question; 14 children indicated that they had no opinion.

^b^ Chi-squared test
