# Supplementary material for: Exploring Perspectives and Barriers in Implementing Home Monitoring for Pediatric Pulmonary Diseases: Mixed Methods Study
Source: JMIR Pediatr Parent. 2026 Oct 20;9:e101452. doi: 10.2196/101452 (PMC13732169; doi:10.2196/101452)
Supplement: Supplementary file 4 [file pediatrics_v9i1e101452_app4.docx]

Multimedia Appendix 4. Median Likert scale score for different types of home monitoring tools for asthma and cystic fibrosis.

| **Type of home monitoring** | **Asthma n = 62**  Median (IQR) | **CF n = 51**  Median (IQR) | **P-value^b^** |
| --- | --- | --- | --- |
| **Smartwatch daytime** |  |  |  |
| Caregivers | 2.0 [1.0-2.0] | 2.0 [1.5-3.0] | .002 |
| Children | 1.0 [1.0-2.0] | 2.0 [1.0-4.0] | <.001 |
| **Smartwatch nighttime** |  |  |  |
| Caregivers | 2.0 [1.0-2.0] | 2.0 [2.0-4.0] | <.001 |
| Children | 2.0 [1.0-2.0] | 2.0 [2.0-4.0] | .007 |
| **Home spirometry** |  |  |  |
| Children | 2.0 [1.0-2.0] | 2.0 [2.0-3.0] | .01 |
| **Cough detector** |  |  |  |
| Caregivers | 2.0 [1.0-2.0] | 3.0 [2.0-3.5] | <.001 |
| Children | 2.0 [1.0-3.0] | 3.0 [2.0-4.0] | .002 |
| **Air quality meter** |  |  |  |
| Caregivers | 1.5 [1.0-2.0] | 2.0 [2.0-2.5] | <.001 |
| Children | 2.0 [1.0-3.0] | 2.0 [2.0-3.0] | .02 |
| **Medication notification** |  |  |  |
| Caregivers | 2.0 [1.0-4.0] | 3.0 [2.0-4.0] | .34 |
| Children | 2.0 [1.0-3.0] | 2.0 [1.0-4.25] | .006 |
| **Questionnaire** |  |  |  |
| Caregivers | 2.0 [2.0-2.0] | 3.0 [2.0-3.0] | .001 |
| Children | 2.0 [2.0-3.0] | 2.5 [2.0-4.0] | .16 |
| **Smart inhaler^a^** |  |  |  |
| Caregivers | 1.0 [1.0-2.0] | 2.0 [2.0-3.0] | <.001 |
| Children | 2.0 [1.0-2.0] | 3.0 [2.0-3.0] | <.001 |

*Participants ranked each monitoring device (1-5). Scoring a 1 participant would very like to use the device. Participants ranking a 5 would really dislike to use the device. All results are presented as median with interquartile ranges.*

**^a^** An inhaler is less used in children with CF

**^b^** Significant difference (P <.05) between groups via Mann-Whitney U test
