# Supplementary material for: Exploring Perspectives and Barriers in Implementing Home Monitoring for Pediatric Pulmonary Diseases: Mixed Methods Study
Source: JMIR Pediatr Parent. 2026 Oct 20;9:e101452. doi: 10.2196/101452 (PMC13732169; doi:10.2196/101452)
Supplement: Supplementary file 5 [file pediatrics_v9i1e101452_app5.docx]

Multimedia Appendix 5. Ability of caregivers and children to notice disease-specific symptoms.

| **Variable – n (%)**  **Caregivers** | Astma n=62 | CF n=51 | P-value^a^ |
| --- | --- | --- | --- |
| Good to very good estimation of symptoms | 37 (59.7%) | 32 (62.7%) | .90 |
| Good to very good estimation shortness of breath | 37 (59.7%) | 42 (82.4%) | .09 |
| Symptoms longer than one day at time of noticing | 28 (45.2%) | 19 (37.3%) | .38 |
| **Children** | *Asthma n=56* | *CF n=48* | *P-value^a^* |
| Good to very good estimation shortness of breath | 35 (62.5%) | 35 (72.9%) | .43 |

^a^ P-value based on the Mann-Whitney U test comparing the full ordinal response distribution (scores 1-5) between the asthma and CF groups.
